# Assessing the impact of the “one-child policy” in China: A synthetic control approach

**DOI:** 10.1371/journal.pone.0220170

**Published:** 2019-11-06

**Authors:** Stuart Gietel-Basten, Xuehui Han, Yuan Cheng

**Affiliations:** 1 Division of Social Sciences, The Hong Kong University of Science and Technology, Hong Kong, PRC; 2 Asian Infrastructure Investment Bank, Beijing, PRC; 3 Population Research Institute, LSE-Fudan Research Centre for Global Public Policy, Fudan University, Shanghai, PRC; University of Louvain, BELGIUM

## Abstract

There is great debate surrounding the demographic impact of China’s population control policies, especially the one-birth restrictions, which ended only recently. We apply an objective, data-driven method to construct the total fertility rates and population size of a ‘synthetic China’, which is assumed to be not subjected to the two major population control policies implemented in the 1970s. We find that while the earlier, less restrictive ‘later-longer-fewer’ policy introduced in 1973 played a critical role in driving down the fertility rate, the role of the ‘one-child policy’ introduced in 1979 and its descendants was much less significant. According to our model, had China continued with the less restrictive policies that were implemented in 1973 and followed a standard development trajectory, the path of fertility transition and total population growth would have been statistically very similar to the pattern observed over the past three decades.

## Introduction

In 2015, China finally ended all one-birth restrictions [1]. The move to a national two-child policy is intended to facilitate a more balanced population development and to counter aging. There is currently a large focus placed on the appraisal of the population control policies (often erroneously thought of as the ‘one-child policy’) imposed in the late 1970s [2]. The world's most comprehensive national-level population control policy has been subject to many criticisms, both domestically and internationally [3, 4]. Sanctioned and unsanctioned instances of forced abortion [5], sterilization [6], and institutional financial irregularities [7] have been identified as bases for criticism. The policies have also been cited as the root cause of other challenges [8], including skewed sex ratios at birth [9], the questionable demographic data because of hidden children [10], and social problems associated with the enforced creation of millions of one-child families (like the social, economic, and psychological plight of couples who lost their only child and are now unable to have more children) [11].

On the other hand, China's population control policies have also been recognized as being effective. This ‘effectiveness’ is based on the estimations that hundreds of millions of births had been ‘averted’ [12] and the penalty of “above-quota-births” was found reducing births in rural China [13]. According to an environmentalist narrative, these births (and the resultant population growth) would have contributed to further climate change [14]. In 2014, for example, *The Economist* labeled the ‘China one-child policy’ as the fourth largest ‘action’ to slow global warming, estimated at 1.3bn tonnes of CO2 [15]. Elsewhere, the popular media, as well as other commentators, regularly espouse a ‘one-child policy' as a panacea to respond to perceived ‘overpopulation' and associated concerns of both an environmental and Malthusian nature. Indeed, UN Resident Coordinator in Kenya, Siddharth Chatterjee, said in 2017 the first annual Africa-China Conference on Population and Development, "China is an example to the rest of the developing countries when it comes to family planning."

These calculations of ‘births averted’ are based on various models, which employ counterfactual history. The estimate of ‘400 million births averted’ is attributed to the one-child population policy [16], which is usually calculated by holding earlier, higher fertility rates constant. Other estimates compared the Chinese experience with either a country or group of countries considered to be similar to China in terms of certain socioeconomic and political indicators. The problem with such counterfactual histories is that they are inevitably subjective and indicators considered did not enter into the model in a systematic way. Contrast to the estimation of 400 million births averted, the effect of the one-child policy is found to be small, especially for the long-run [17], which was attributed to the aggressive family planning program in the early 1970s [18] based on the findings that the birth rate of 16 countries with similar birth rates to that of China in 1970 declined significantly after 1979 and even sharper than what was observed in China [19].

To evaluate the impact of China’s population control policies, we employ the Synthetic Control Method where we compare China to a constructed ‘synthetic’ control population, which shares similar features with China during the pre-intervention periods. This innovative data- and math-driven methodology is used extensively in many disciplines, including public health [20], politics [21], and economics [22]. One of the caveats of our paper is that we cannot single out the ‘cohort’ effects. In addition to the socio-economic factors, the decline of TFRs might partially be the result that females entering childbearing age in 1970s did not think giving more births is “fashionable” compared to those who entered childbearing age in 1950s. Such mindset changes have been observed in Brazil [23]. Unfortunately, our approach cannot differentiate the cohort effect from the impact of social-economic factors. We have to bear in mind this caveat in the following analysis.

In the case of China, the first intervention (or ‘shock’) we seek to evaluate is the ‘Later-Longer-Fewer Policy’ introduced in 1973 [7]. Under this policy, a minimum age of marriage was imposed, as well as mandatory birth spacing for couples and a cap on the total number of children [24]. The rules were differentiated for men and women in rural and urban areas. Also, like the case in other countries, widespread contraception (and free choice) was introduced, coupled with large-scale education on family planning [25]. The second ‘shock’ is the ‘One-Child Policy' introduced in 1979, where a one-child quota was strictly enforced. Following initial ‘shock drives' of intensive mass education, insertion of IUDs after the first birth, sterilization after the second birth, and large-scale abortion campaigns, the policy quickly became unpopular and was reformed in 1984 and onwards, creating a very heterogeneous system [26]. Despite the series of reforms, the majority of couples in China were still subject to one-child quotas in the 1980s and 1990s.

## Institutional Background

With high birth rates in the 1970s, the Chinese government had grown increasingly concerned about the capacity of existing resources to support the ballooning population. In response, from 1973, the Chinese government widely promoted the practice of ‘later-longer-fewer’ to couples, referring respectively to later marriage and childbearing, longer intervals between births, and fewer children. Rules were more severe in urban areas where women were encouraged to delay marriage until the age of 25 and men at 28 and for couples to have no more than two children. In the rural areas, the age of marriage was set at a minimum of 23 for women, and 25 for men and the maximum family size was set at three children. Birth control methods and family planning services were also offered to couples. The policy at the time can be considered “mild” in a sense that couples were free to choose what contraceptive methods they would use and the policy on family planning was more focused on the education of the use of contraceptives [27].

However, such mild family planning program was deemed insufficient in controlling the population, since it would not be able to meet the official target of 1.2 billion people by 2000 despite the large decrease in the total fertility rate (TFR) in the late 1970s. In 1979, the government introduced the One-Child Policy in the Fifth National People’s Congress, a one-size-fits-all model and widely considered the world’s strictest family planning policy. Some exemptions were allowed, and a family could have more than one child if the first child has a disability, both parents work in high-risk occupations, and/or both parents are from one-child families themselves. The State Family Planning Bureau aimed to achieve an average of 1.2 children born per woman nationally in the early and mid-1980s [27].

From 1980 to 1983, the one-child policy was implemented through "shock drives" in the form of intensive mass education programs, IUD insertion for women after the first birth, sterilization for couples after the second birth, and abortion campaigns for the third pregnancy [27, 28]. Policies were further enforced by giving incentives for compliance and disincentives for non-compliance, though these varied across local governments [27]. Liao [29] identified the following as the usual benefits and penalties at the local level. Families with only one child can obtain benefits like child allowance until age 14; easier access to schools, college admission, employment, health care, and housing; and reduction in tax payments and the opportunity to buy a larger land for families in rural areas. Penalties for having above-quota births, on the other hand, include reduction in the parents’ wages by 10 to 20 percent for 3 to 14 years, demotion or ineligibility for promotion for parents who work in the government sector, exclusion of above-quota children to attend public schools, and, in rural areas, a one-time fine which may account for a significant fraction of the parents’ annual income.

The tight one-child policy was met by resistance, and the government allowed more exemptions [27]. Exemptions were drafted at the local level as the Chinese Communist Party’s Central Committee took into account the diverse demographic and socioeconomic conditions across China [30]. In 1984, the program allowed two births per couple in rural areas if the first child is a girl or if the family is from a minority ethnic group, but this was done only in six provinces. One significant change in the family planning policy is that couples with one daughter in rural areas could have a second child after a certain interval, which ranges from four to six years, and this was fully implemented in 18 provinces by the end of 1989. The performance of local cadres was also evaluated with family planning activity as the top criterion [27]. The stringency of the one-child policy was further moderated amid China’s commitment to the International Conference on Population Development held in Cairo in 1994. In 1995, the family planning program changed its stance from being target-driven to client-centered in adherence to international reproductive health standards. More attention was given to individual contraceptive rights, and the government allowed couples to choose their contraceptive method with the guidance of the professional and technical staff [22].

Throughout the 1990s, provinces amended their own regulations about the exemptions under the guidelines of the State Family Planning Commission, now the National Population and Planning Commission [30]. According to Gu et al. [30], the provincial-level exemptions on allowing more than one child in a family can be classified into four broad groups: (1) gender-based and demographic (if the couple living in a rural area had the only daughter, or they belong to one-child family themselves); (2) economic (if the couple work in risky occupations or have economic difficulties); (3) political, ethical, and social (if the couple belong to a minority ethnic group, the man is marrying into a woman’s family, the family is a returning overseas Chinese, or the person has the status of being a single child of a revolutionary martyr); and (4) entitlement and replacement (if the couple’s first child died or is physically handicapped, the person who is divorced or widowed remarries, or the person is the only productive son in a family of multiple children in the rural area).

While the central government had asserted that population control remains a basic state policy, it hardly implemented a uniform set of rules across the country, hence the varying exemptions across localities [30]. This was until the Population and Family Planning Law of 2001 was put into effectivity. The law summarized the rights and obligations of Chinese citizens in family planning and served as the legal basis for addressing population issues at the national level. This law still promoted the one-child policy, but couples were given more reproductive rights, including the right to decide when to have children and the spacing between children if having a second child is allowed, as well as the right to choose contraceptive methods. It also discussed the imposition of social compensation fees for those who violated the law, which will be collected by local governments and family planning officials [27].

The one-child policy was further loosened in 2013 when it was announced that two children would be allowed if one parent is an only child [31]. Basten and Jiang [32] summarized the popular views on the issues that can be addressed by this policy shift: skewed sex ratio at birth, projected decline of the working-age population, large number of couples who were left childless because of the death of their only child, and evasion and selective enforcement of fines for out-of-quota and unauthorized births. They, however, argued that this change in the one-child policy could only have minimal impact on the aging population and shrinking workforce because of fertility preferences to have only one child and a smaller likelihood of these births to occur.

It was announced in October 2015 that the one-child policy would be replaced by a universal two-child policy. Driven by some evidence that this relaxation of the policy has not achieved a significant birth boosting effect, the Chinese government has started in 2018 to draft a proposed law that will remove all the limits on the number of children families can have [33].

## The Synthetic control method

In this paper, we aim to assess the impact of the 1973 and 1979 family planning policies and to explain why there was no significant rise in the fertility rate observed after the birth control policy was relaxed in 2015. For this purpose, we use the Synthetic Control Method proposed by Abadie et al. [20, 21] to the context of fertility behavior. The nature of the synthetic control method is to find countries with very similar fertility and other fertility-related demographic and socioeconomic features as China before the policy intervention by giving more weights to countries with the most similarities. For the post-intervention period, the fertility rate of similar countries with their corresponding weights is used to construct the synthetic China TFR, which represents the fertility rate if there were no policy intervention. The difference between the synthetic TFR and the observed TFR after the intervention is the impact of the policy. We formulate the relationship between the with- and without-intervention TFR as follows:

$${TFR}_{it}^{73-79}={TFR}_{it}^{No\;73\;policy}+\alpha_{it}^{73}D_{it}^{73},\;where\;D_{it}^{73}=1,\;for\;1973\leq t<1979;otherwise=0;$$ (1)

$${TFR}_{it}^{79\;onward}={TFR}_{it}^{with\;73\;but\;no\;79\;policy}+\alpha_{it}^{79}D_{it}^{79},\;where\;D_{it}^{79}=1,\;for\;t\geq 1979;otherwise=0.$$ (2)

${TFR}_{it}^{73-79}$ are the total fertility rate of country *i* after 1973 but before 1979 while ${TFR}_{it}^{79\;onward}$ are the total fertility rate of country i in time after 1979. ${TFR}_{it}^{No\;73\;policy}$ and ${TFR}_{it}^{with\;73\;but\;no\;79\;policy}$ represent the TFRs assuming that there were no interventions and the TFRs assuming that there was only the 1973 intervention, respectively. $D_{it}^{73}$ and $D_{it}^{79}$ are dummy variables that take the value of one if country *i* is exposed to the respective intervention, which depends on whether the time *t* is pre- or post- the policy year. $\alpha_{it}^{73}$ and $\alpha_{it}^{79}$ capture the effect of the interventions in 1973 and 1979, respectively.

There are altogether *J*+1 countries and *T* time periods. For simplicity, we use ${TFR}_{it}^{N}$ to represent either ${TFR}_{it}^{No\;73\;policy}$ or ${TFR}_{it}^{with\;73\;but\;no\;79\;policy}$ in the following deductions. Suppose that ${TFR}_{it}^{N}$ is given by a factor model:

$${TFR}_{it}^{N}=\delta_{t}+\theta Z_{it}+\lambda_{t}\mu_{i}+\varepsilon_{it},$$ (3)

where *δ*_*t*_ is constant across all countries and only varies with time. *Z*_*i*_ is a vector of observable variables that we believe affect the fertility rate but is not affected by the intervention policy. *μ*_*i*_ represents an unobservable factor affecting fertility rate and varies across countries. *ε*_*it*_ is the error term with zero means. To simplify, we equate *i* = 1 for China and *T*_0_ (either 1973 or 1979) is the policy intervention year with 1≤*T*_0_≤*T*.

We sum up the left-hand side and the right-hand side of Eq (3) for each period *t* before the intervention $\left({TFR}_{it}={TFR}_{it}^{N}\right)$ in all countries except China using different weights, which can be expressed as:

$$\sum_{j=2}^{J+1}w_{j}{TFR}_{jt}=\delta_{t}+\theta_{t}\sum_{j=2}^{J+1}w_{j}Z_{j}+\lambda_{t}\sum_{j=2}^{J+1}w_{j}\mu_{j}+\sum_{j=2}^{J+1}w_{j}\varepsilon_{it},\;where\;w_{j}\geq 0\;and\;w_{2}+\cdots +w_{J+1}=1.$$ (4)

The optimal $w_{j}^{*}$ achieves the following target:

$$\sum_{j=2}^{J+1}w_{j}^{*}\;{TFR}_{jt}={TFR}_{1t}\;and\;\sum_{j=2}^{J+1}w_{j}^{*}\;Z_{jt}=Z_{1t}\;for\;each\;t\;where\;t\leq T_{0}.$$ (5)

The optimal $w_{j}^{*}$ are the weights applied to replicate China’s fertility rate and other characteristics by using the fertility rates and other characteristics of all the other countries.

Therefore, for the period after intervention, the impact of the intervention can be estimated by:

$${\hat{\alpha }}_{it}^{73}={TFR}_{1t}-\sum_{j=2}^{J+1}w_{j}^{*}\;{TFR}_{jt},\;for\;1973\leq t<1979;$$ (6)

$${\hat{\alpha }}_{it}^{79}={TFR}_{1t}-\sum_{j=2}^{J+1}w_{j}^{*}\;{TFR}_{jt},\;for\;t\geq 1979.$$ (7)

We obtain the optimal $w_{j}^{*}$ by minimizing the distance between ‖*X*_1_−*WX*_0_‖ where:

$$X_{1}={\left(Z_{1}^{\prime },{\bar{TFR}}_{1}^{\frac{1}{T_{0}}}\right)}^{\prime };$$ (8)

$$X_{j}={\left(Z_{j}^{\prime },{\bar{TFR}}_{j}^{\frac{1}{T_{0}}}\right)}^{\prime },\;which\;is\;the\;jth\;column\;of\;X_{0}.$$ (9)

As reflected in the above procedure, the core of this method focuses on finding the combination of countries that collectively resemble China before the intervention. The model automatically assigns different weights to different countries in such a way that the distance between the actual and synthetic China before the policy intervention will be minimized in terms of fertility rate and other related characteristics. The optimal weights then are applied to the other countries for the post-intervention period to obtain Synthetic China without either the 1973 intervention or the 1979 intervention.

## Data

The next step is to decide what variables should be included in vector Z. We chose to include the childbearing age, life expectancy at birth, and sex ratio of male to female between 0 and 4 years old as the non-economic variables. The childbearing age affects the mothers’ age-specific fertility intensity and the total fertility rate [34, 35]. With the maximum fertility age being certain, higher childbearing age might imply lower TFR. The life expectancy at birth is related to age-specific mortality. With a lower mortality rate, fewer births are required to obtain a desired number of children. For example, as observed by Galor [36], the TFR declined while the life expectancy improved in Western Europe in the past half-century. The sex ratio of male to female represents the inner-gender competition. A higher sex ratio of male to female implies higher competition among males, so it is more rewarding for females to delay marriage and to give birth in exchange for opportunities to obtain a better match with males. Using data from England and a generalized linear model, Chipman and Morrison [37] confirmed the significant negative relationship between the sex ratio of male to female and birth rate, especially for the three age groups of females at 20–24, 25–29, and 30–34 years old.

The other group of variables included in vector *Z* is economic variables, such as GDP per capita and years of schooling. The New Home Economics approach [38] emphasizes the negative relationship between income and fertility rate through the role of the opportunity cost of parenting time. The model suggests that more children will consume more parenting time, which could otherwise be used to generate more income. Galor and Weil [39] strengthened the reasoning by arguing that the increase in capital per capita raises women’s relative wages because the complementary effect of capital to female labor is higher than to male labor. The increase in women’s relative wage raises the cost of children. Because of the resulting smaller population effect, the lower fertility further raises the GDP per capita. In addition to the parenting opportunity cost, the economic development might result in fertility declines through two other channels:(1)With economic development, the living standards improved and the mortality rate decreased so that parents can have the same desirable living kids with fewer births; and (2) With the economic development, people have more tools to save, for example, the pension system, which reduces the needs of having more offspring to finance the retirement. The relationships between the macro-economy and the fertility patterns are documented for China [40, 41, 42]. The years of schooling also affects fertility through the opportunity costs channel. Higher education is associated with higher productivity, which would induce the higher opportunity cost of raising children.

Our analysis uses the TFR data in the period of 1955–1959 from the United Nations’ World Population Prospects (WPP) and the annual TFR data in 1960 to 2015 from the World Bank’s World Development Indicators (WDI) except for the following five economies. For Curaçao, Luxembourg, Serbia, Seychelles, and Taiwan, we use the UN’s WPP data in the entire period of 1955 to 2015. Like in the TFR data, we use the life expectancy at birth data in the period 1955–1959 from the UN’s WPP data, while annual life expectancy data in 1960 to 2015 is obtained from the WDI, except for the following four economies. For Curaçao, Serbia, Seychelles, and Taiwan, we use the UN’s WPP in the entire period of 1955 to 2015. The whole data series of the male-to-female ratio of the population aged 0–4 years old are obtained from the UN. We use the expenditure-side real GDP at chained PPPs and the size of population data from the Penn World Tables 9.0 (PWT 9.0) to calculate the GDP per capita and get its natural logarithm. The average years of schooling data obtained from the Barro-Lee Database is used to measure the average level of education in a given country. Historical schooling data are only available at five-year intervals, so we apply a linear interpolation method to infer the annual data from 1950 to 2010. The average childbearing age data are from the UN’s WPP in the entire period of 1955 to 2015. Additionally, all WPP data, except the male-to-female ratio, are only available at a five-year interval, so we also employ the linear interpolation method to get the annual estimates.

The original dataset consisted of 184 countries, but after removing the countries with missing data for the needed variables from 1955 to 2010, only 64 countries remained in the final dataset for the analysis, including China. The final list of countries included in the analysis is provided in Table A in S1 File.

## Empirical result

For simplicity, we label synthetic China as *Synth*China, whose characteristics are constructed using the values of the other countries and the countries’ corresponding weights. We present the average values of our target variable TFR and fertility-related variables for *Synth*China and our comparator in Table 1. The column on China shows the actual numbers for China, while the column on *Synth*China displays the values for the counterfactual *Synth*China for the pre-1973 period and pre-1979 and post-1973 period. For comparison purposes, we also include the average values of all countries in the sample as our comparator to show how different it would be between actual China and the whole sample in the absence of synthesizing. Looking at the pre-1973 period, *Synth*China has the same average TFR of 5.85 as actual China, while our comparator has an average of 4.71. For the remaining variables, the values of *Synth*China are all closer to that of actual China than those of our comparator, which indicates that *Synth*China resembles actual China not only in terms of TFR but also in terms of other fertility-related characteristics. Looking at the pre-1979 and post-1973 period, the TFR of *Synth*China is again almost the same as that of actual China.

**Table 1:** Averages of Pre-intervention characteristics of China, SynthChina, and the comparator.

	China (Actual)	*Synth*China (Simulated for Pre-Intervention Periods)	Our Comparator (All countries in the sample)
*Pre- 1973*:			
TFR	5.85	5.85	4.71
Male to Female (0–4)	1.05	1.05	1.04
Childbearing Age	29.76	29.74	28.65
Ln GDP per Capita	7.12	7.82	8.66
Life Expectancy at Birth	56.44	56.50	63.18
Years of Schooling	3.67	3.27	5.13
*Pre-1979 but Post- 1973*			
TFR	3.59	3.60	3.89
Male to Female (0–4)	1.06	1.06	1.04
Childbearing Age	29.17	29.13	28.31
Ln GDP per Capita	7.26	8.25	8.87
Life Expectancy at Birth	64.15	63.41	65.59
Years of Schooling	4.66	6.31	5.91

Note: For the pre-1973, the pre-intervention period for TFR is 1955–1973 while for the others are 1965–1973.

All the other variables of *Synth*China are more comparable to actual China than to our comparator, except for average years of schooling. The significant difference (1.65 years) in years of schooling for the period of 1973–1979 between China (4.66 years) and the Synthetic cohort (6.31 years) is mainly due to the school-year-reduction-reform to taken by the Chinese government during the cultural revolution period (1966–1976). The original 6 years of primary schooling, 3 years of middle school, and 3 years of high school (6-3-3) for the pre-1966 periods were reduced to 5-2-2, respectively [43]. That means the same length of years of schooling represented higher accomplishment in terms of a diploma during 1966–1976. Five years of schooling in this period indicated completion of preliminary school while it used to represent the unaccomplished preliminary school. Most countries included in the studies adopted the 12-year schooling system. If we measure the accomplishment of education by using the relative years of schooling, which is to scale down by the years required for completion of high school—52% (4.66/9) for actual China and 53% (6.31/12) for Synthetic cohort—we would have quite close level of relative years of schooling between China and the Synthetic cohort. Additionally, the difference in years of schooling between actual China and the Synthetic cohort was not as significant for the pre-1973 intervention period (1965–1973) as for the pre-1979 and post-1973 period is because even the implementation of the school-year-reduction-reform was started from 1966 it requires five years for the effects to be fully materialized. The education system was changed back to 6-3-3 system after 1976.

In the following simulation, we use the periods 1973–1979 and 1980–2015 as the post-intervention periods to quantify the impact of the first and second shocks, respectively.

**Fig 1:**
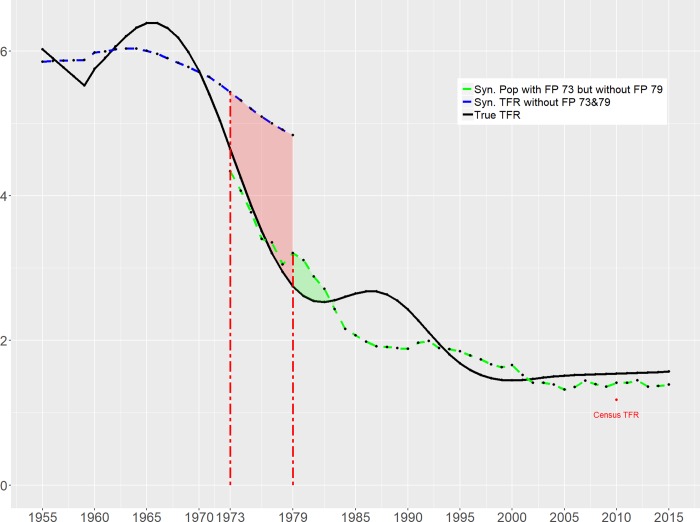
Time trend of ‘actual’ and ‘synthetic’ Total Fertility Rate for China, 1955–2015.

The TFR simulated for *Synth*China assuming without the 1973 shock, with the 1973 shock but without the 1979 shock, and the actual TFR are plotted in Fig 1. The dashed blue line represents synthetic China's simulated TFR in the period 1955–1979 assuming without 1973 shock. The gap between the *Synth*China and actual China (represented by the solid black line) between 1973 and 1979 is the reduction in the TFR caused by the 1973 intervention. The dotted green line is the TFR of *Synth*China estimated for the period 1973–2015 with the period 1973–1979 as the pre-intervention period set to search for the optimal weights, which is to find the best comparable countries with fertility behaviors like China with 1973 shock but without 1979 shock. The simulated TFR for periods after 1979 is supposed to represent the TFR of China with the 1973 policy but without the 1979 policy. Contrary to the commonly claimed radical effect, the “One-Child” policy in 1979 only induced a small dip in the TFR.

As shown in Fig 1, the TFR in synthetic China is already well above the real TFR, even before the 1973 shock. The reason is that the best fit found by the algorithm cannot match the whole pattern of actual TFR (a complete overlap of actual and simulated China) for the pre-intervention periods, especially for the pre-1973 period (blue line). As shown in section 3, the target function for optimization is ‖*X*_1_−*WX*_0_‖, which measures the distance between the mean of actual China and *Syn*China without the policy of 73&79 for years before 1973. When the pattern of actual TFR is not well regulated, the simulated TFRs for the pre-1973 periods cannot match actual China for each year of the time series but to match on the average over the periods. It is why for pre-1960 periods, the blue line is above the black line while for the periods of1960-1970, the blue line is below the black line. Our conjecture on the reason for the irregular pattern of actual China in pre-1973 periods is that the government had been in a population policy struggling during this period [44] and the after-effect of the great fluctuations caused by China's Great Leap Forward famine (1958–1962). For example, right after the promotion of birth control policy in 1957, the birth control was catalyzed as anti-government in 1958. Not until 1962, birth control was encouraged again. Such changes of direction of the policy were very hard to simulate by finding the best comparable. Additionally, we identify the official announcement of "Later-Longer-Fewer Policy" in 1973 as the "shock." The informal introduction of such an idea started from 1971 when the encouragement of birth control was included as a "national" strategic policy. But only until 1973, the policy was announced officially with details. This explains why the SynthChina with FP 73&79 is already above actual China in 1973.

One interesting observation is that the TFR of *Synth*China with 1973 shock but without 1979 shock is lower than the observed TFR since 2003. Combining with the fact that the TFR reported in the Sixth Census in 2010 is lower than the TFR of *Synth*China, this appears to be providing indirect evidence on the common suspicion that the statistics on fertility rate might be “too low” and therefore the fertility effect of the 1979 policy could have been overstated.

**Fig 2:**
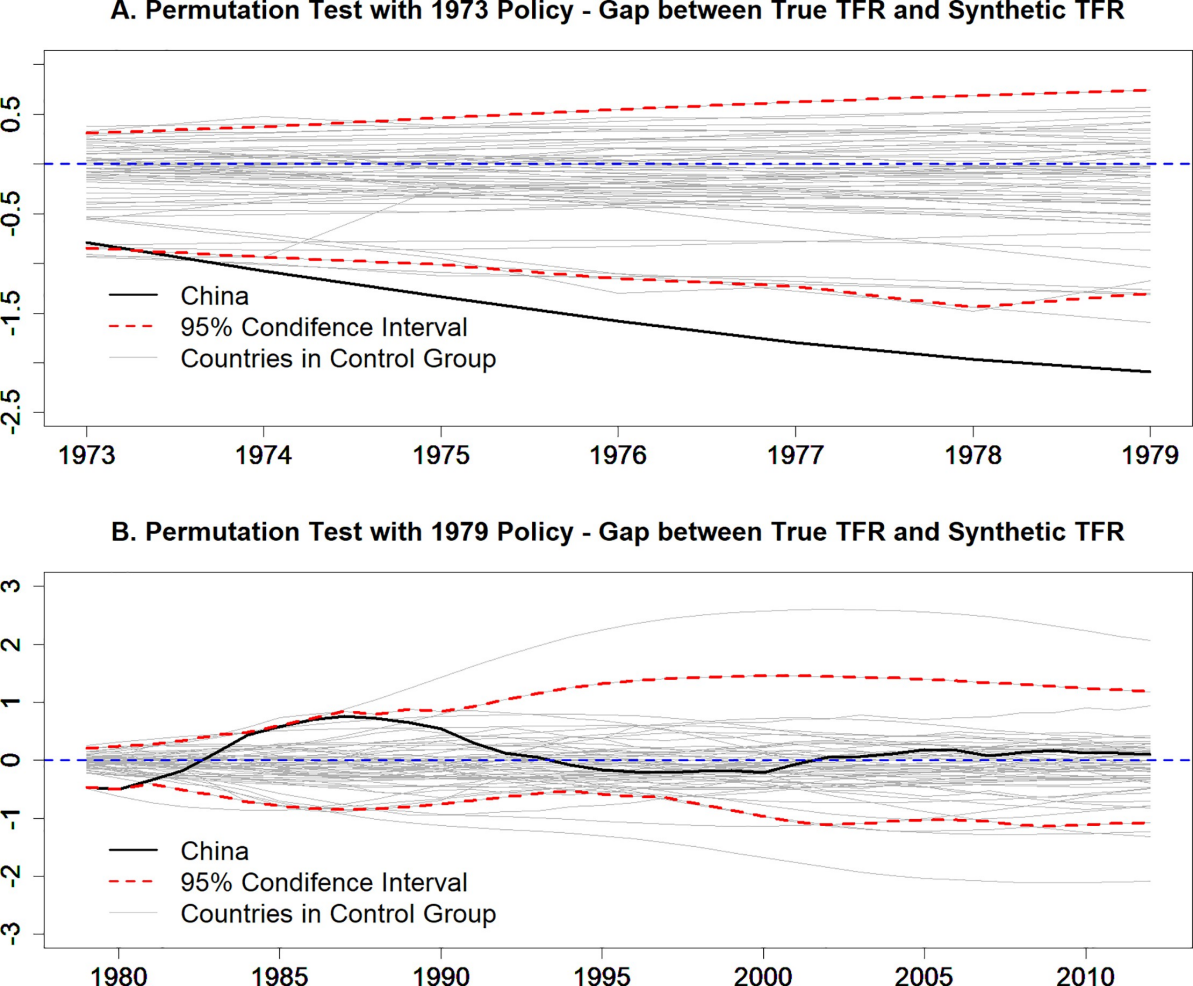
Robustness permutation test on the significance of the 1973 and 1979 policy impact. (A)Permutation test with 1973 policy–gap between true TFR and synthetic TFR. (B) Permutation test with 1979 policy–gap between true TFR and synthetic TFR.

Next, we apply the permutation test to evaluate the significance and robustness of the estimations. To do this, we produce a simulated sample of 500 countries by randomly drawing with replacement from the actual sample of 63 countries with China being excluded. Each country is treated as if it were China and is subjected to the 1973 and 1979 shocks. We construct the synthetic TFRs by following the same procedure carried out for *Synth*China. For each year, we calculate 500 simulated gaps between actual and synthetic TFRs, as shown in Fig 2. The gaps for the simulated countries are represented by the grey lines, while the 95% confidence intervals by the red lines. The solid line denotes the gap between actual and *Synth*China, which is well below the lower bound of the 95% confidence interval from 1973 to 1979, indicating a significant reduction impact from the 1973 shock (Fig 2). Meanwhile, the TFR gap between actual and *Synth*China stays within the confidence interval from 1980 onwards, implying that the 1979 shock had no significant impact (Fig 2).

Population projection is carried out by using *Spectrum 10*, wherein the actual TFR was replaced by the synthetic TFR from 1979 to 2015.

**Fig 3:**
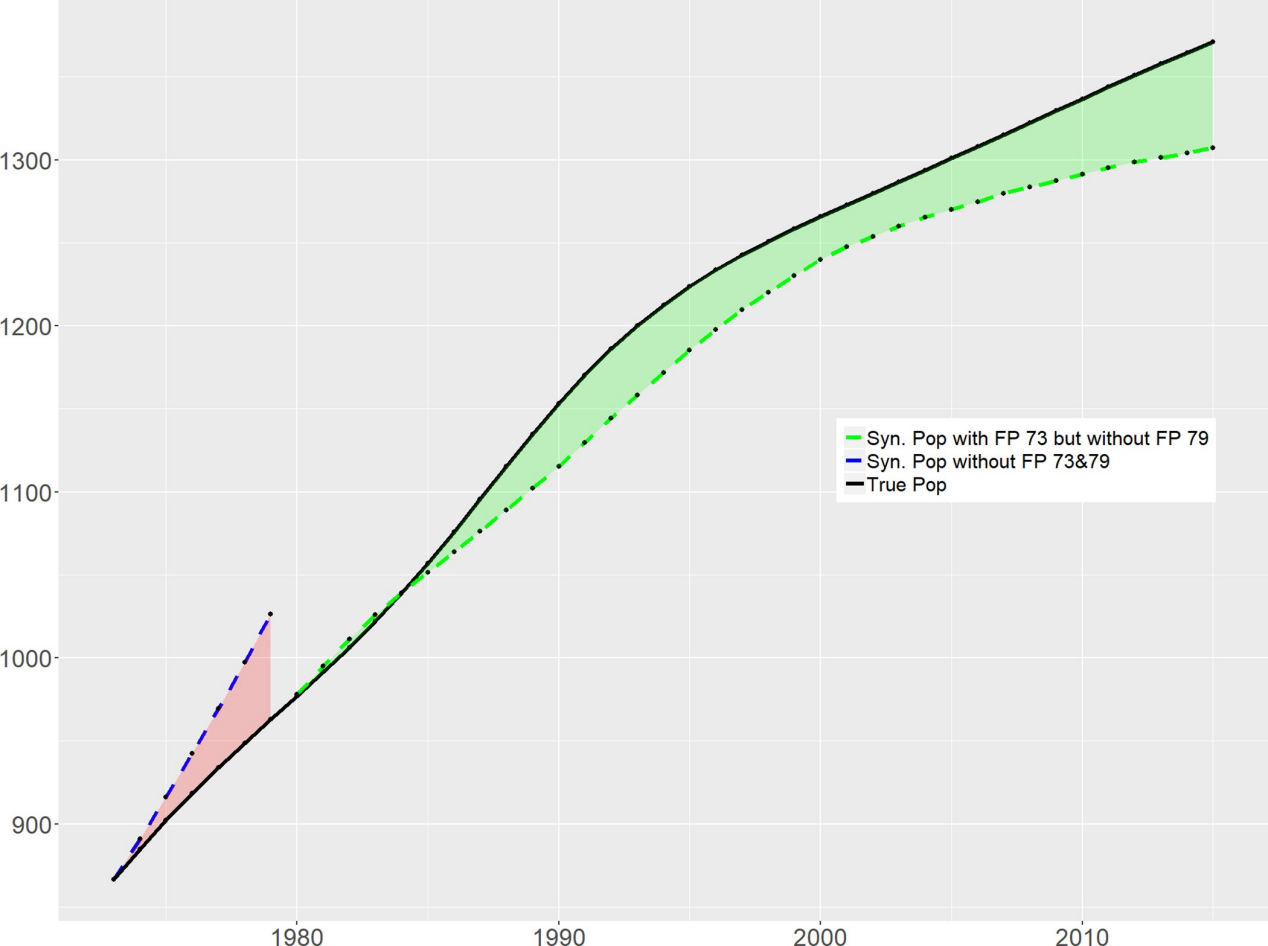
Time trend of population sizes under ‘true’ and ‘synthetic’ TFR for China, 1955–2015.

As Fig 1 and Fig 2 show, had China not implemented its later-longer-fewer set of population control measures in 1973, the fall in TFR would have been much shallower. Translating this into total population, this would amount to a difference of around 85 million by the end of the 1970s (Fig 3). The impact of the second ‘shock,' namely the introduction of the stricter control measures in 1979, appears to be much more muted. While there are differences in the 1980s as a result of the reform involving the regulation on marriage age, the TFR for *Synth*China and actual China are broadly in sync from the early 1990s. In terms of total population difference, *Synth*China is some 70 million *lower* than actual China by 2015, as shown in Fig 3. As discussed above, this puzzling outcome of the second shock might be due to the overstating tendency of the fertility statistics. Based on the permutation tests shown in Fig 2, we can conclude that the 1973 policy significantly reduced the population by 85 million, while the 1979 policy does not have a statistically significant impact.

Furthermore, we use a bootstrap strategy to get the confidence interval for the population estimates assuming without the shock of 1973 policy. We randomly drew 500 sub-samples with the size of 90% of the original sample without replacement. For each sub-sample, we repeated the synthetic control approach to search for the best synthetic China in terms of TFR. Among the 500 subsamples, two samples cannot converge. Therefore, in the end, we have 498 Synthetic China. We further get the 5% lower and upper bounds of TFRs among simulated Synthetic China. Building on the 5% lower and upper bounds of TFRs, we further calculate the resulted population, with which to compare the actual population and get the corresponding reduced population. The lower and upper bounds of the reduced population serve as the 90% confidence interval of Synthetic China in terms of the population without 1973 policy shock. The corresponding reduction of the population associated with the 1973 policy is between 60 and 94 million.

As shown in Table 2, the countries used to construct *Synth*China differed significantly between the 1973 and 1979 shocks. Before the 1973 shock, the greatest contribution was made by India (with a weight of 36.9%), a country that implemented a weaker family planning system and was characterized by high fertility throughout the 1970s [45]. Jordan, Thailand, Ireland, Egypt, and Korea came as the second to the sixth most comparable countries to China. All of them, except Ireland, had family planning policies. Jordan started family planning measures in the 1980s [46]; Thailand had done three rounds of family planning measures starting from 1963 to 1980 [47]; Egypt implemented three rounds of family planning measures in 1966, 1970, and 1979 [48]; and the family planning policy started in Korea in 1961 and lasted until the 1980s [49]. Even without any institutional background information, the synthetic control model has been able to select countries with family planning programs automatically.

**Table 2:** Countries resembling China with significant weights.

Pre-1973 Period		Pre-1979 but Post- 1973 Period	
India	36.9%	India	4.1%
Ireland	10.3%	Jordan	1.4%
Jordan	21.1%	Korea	75.2%
Korea	1.2%	Thailand	16.0%
Thailand	15.2%		
Egypt	1.3%		

Note: For the pre-1973 period, altogether, 57 countries were used to construct the *Synth*China. Here we only present countries with weights higher than 1%. For the pre-1979 but post- 1973 period, altogether, 21 countries were used to construct the *Synth*China. Here we only present countries with weights higher than 1%.

In the period 1973 to 1979, Korea overtook India as the country that most resembled China (75.2%). While the GDP per capita was considerably different between these two countries in this period (even in the current period), in the 1980s, they shared similarities in terms of the other variables not included in the model, including the GDP growth rate and the presence of an authoritarian political regime [50, 51]. Furthermore, the Korean family planning system was extraordinarily comprehensive and was founded on new social norms around family size, as well as the development of rural areas in general [52]. Thailand still played an important role with a contribution of 16% to *Synth*China.

## Robustness check

We further carried out several robustness checks by including the add-on policy intervention or altering the data coverage.

We examined first the impact of the commonly acknowledged temporary relaxation of the one-child policy during the late 1980s until the beginning of 1990s by using 1991 as another intervention year (Table B and Fig A in S1 File). No significant impact was found.

A second robustness check done was performed by extending the coverage of the dataset. The baseline dataset of 64 countries used in the analysis was constructed by excluding countries with any missing value for the input and output variables from 1955 to 2010. Therefore, there is a possibility that countries sharing great similarities with China were excluded because of unavailable GDP per capita data in 1955 and onwards. The GDP per capita data were obtained from PWT 9.0, which is mostly accepted as one of the most reliable and complete sources of GDP data, especially when comparison across countries is required. To examine whether such exclusions would alter our conclusion, we revised our data construction by relaxing the time coverage requirement and allowing an unbalanced dataset for each shock. That is, if the input variables of a country for the required years by the Synthetic Control Method were available, we included it in the dataset. For example, countries previously excluded from our baseline model because of missing data on GDP per capita from 1955 to 1964 were included for assessing the impact of 1973 shock, and the availability of the GDP per capita data was only required from 1965 to 1973. It resulted in a dataset containing 103 countries for the 1973 shock and 123 countries for the 1979 shock (Tables C and D in S1 File). Consistent with our baseline results, there was a significant decline in the TFR associated with the 1973 shock but insignificant impact with the 1979 shock (Table E and Fig B in S1 File).

The final main robustness check done is restricting the coverage of countries in the dataset. We selected 25 countries as a focus group that had been subjectively recognized by previous literature as having similar fertility behavior as China (Table F in S1 File). The focus group dataset with available data consisted of 17 countries for the 1973 shock and 20 countries for the 1979 shock. India, Indonesia, and Thailand were selected for *Synth*China in evaluating the 1973 shock and Korea, and Thailand was selected for *Synth*China in evaluating the 1979 shock, which was fewer than in our baseline analysis (Table G in S1 File). Interestingly, the permutation test showed that even for the 1973 shock, the gap between the TFR of *Synth*China and actual TFR is located within the 95% interval. This indicates the insignificant impact of the 1973 shock. However, since there were only 16 countries used to do the random draw for the 500 paths, the variation contained in the permutation test is very limited, which weakened the reliability of the test (Fig C in S1 File). The lower bound of the 95% confidence interval was dominated by Korea. Korea experienced a much sharper decline in TFR in the 1970s. Excluding Korea, China had the largest gap in the TFR.

As a robustness check, we also replace the TFRs used in our analysis with the UN-provided interpolated annual TFRs. The result is consistent with our baseline findings (see Table H and Fig D in S1 File).

## Limitations and conclusions

Of course, our study has various limitations. Firstly, from a data perspective, it is arguable that the veracity evidence derived for China–and, indeed, reconstructed for other countries–over the past seven decades is to be open to interpretation. This potential challenge is acknowledged and would, indeed, affect any and all studies of Chinese population history. However, the main argument of the likely impact of these two shocks still holds. Secondly, by considering China as a national unit, we do not disaggregate and consider the impact of the interventions (and policy differentials) at the sub-national unit. For example, it may be that the 1979 intervention had a more significant impact in one province than in others, dependent on the social and economic conditions of that region, coupled with the particular ‘history’ of birth control policies there. By considering only the aggregate level, we lose this granularity. Such an exercise would be a fruitful future avenue of research. The final criticism is a more holistic one. Is the size, complexity, the political, and economic system of China so unique that it is possible to create a ‘synthetic China’ at all? For sure, China is ‘different’ to most, if not all, countries of the world. However, the principle of the synthetic control approach is simply to draw similarities from other places if and where they exist. In this way, such an approach is more systematic, transparent, and viable than simply drawing on a single country comparator or a basket of other regions. Indeed, it could be argued that all possible units of analysis (countries, regions, towns) are ‘unique’ in their own way.

In this paper, we used the synthetic control method to assess the impact of the "One-Child" policy in China. Our findings strongly suggest that had China followed a standard development trajectory combined with the continuation of its comprehensive population control policies introduced in 1973 (‘later-longer-fewer'), the decline in the TFR and hence total population size would have been similar under the conditions of the stricter one-child policy and its various reforms thereafter. While the policies implemented in 1973 were restrictive in terms of spacing, timing and the quantum total number of children, and were also stricter than almost any other contemporary family planning program, they were, undoubtedly, less restrictive than what followed.

The implications of this study are two-fold. Firstly, by suggesting that the impact of the birth control policies may have been exaggerated in the past, we can better understand why the response to their *relaxation* has been relatively muted–or, at least, well below popular expectation. Secondly: it is impossible to ignore the fact that the strict birth control policies introduced in 1979 brought with them numerous negative and possibly unforeseen consequences. As well as the sanctioned activities and corrupt abuses which occurred within the birth control policy framework, the policies have been linked to the highly skewed sex ratio [53], the presence of millions of *shidu fumu* families who have lost their only child [54] as well as other challenges in both the development of family systems and individual behavior. The long-term psychological consequences of prioritizing one-child families have yet to be fully explored, not least in the context of possible efforts to spur childbearing in the future.

In this context, our analysis suggests that the population control policies implemented from 1979 have no significant demographic effect compared to a looser operationalization of population control and economic development. An important lesson for other countries that are planning to introduce population controls: the stricter controls might not be the effective one.

## Supporting information

S1 File Appendix. (DOCX)

S2 File Program and data. (RAR)
