# Supplementary material for: Assessing the impact of the “one-child policy” in China: A synthetic control approach
Source: PLoS One. 2019 Nov 6;14(11):e0220170. doi: 10.1371/journal.pone.0220170 (PMC6834373; doi:10.1371/journal.pone.0220170)
Supplement: S1 File — (DOCX) [file pone.0220170.s001.docx]

Appendix

**Table A. Country List to Calculate Weights in Constructing** *Synth*China

| 1 | Argentina | 17 | Egypt | 33 | Kenya | 49 | Portugal |
| --- | --- | --- | --- | --- | --- | --- | --- |
| 2 | Australia | 18 | El Salvador | 34 | Korea, Rep. | 50 | South Africa |
| 3 | Austria | 19 | Finland | 35 | Luxembourg | 51 | Spain |
| 4 | Belgium | 20 | France | 36 | Malawi | 52 | Sri Lanka |
| 5 | Bolivia | 21 | Germany | 37 | Malta | 53 | Sweden |
| 6 | Brazil | 22 | Greece | 38 | Mauritius | 54 | Switzerland |
| 7 | Canada | 23 | Guatemala | 39 | Mexico | 55 | Taiwan |
| 8 | Chile | 24 | Honduras | 40 | Netherlands | 56 | Thailand |
| 9 | China | 25 | Iceland | 41 | New Zealand | 57 | Trinidad and Tobago |
| 10 | Colombia | 26 | India | 42 | Nicaragua | 58 | Turkey |
| 11 | Congo, Dem. Rep. | 27 | Ireland | 43 | Norway | 59 | Uganda |
| 12 | Costa Rica | 28 | Israel | 44 | Pakistan | 60 | United Kingdom |
| 13 | Cyprus | 29 | Italy | 45 | Panama | 61 | United States |
| 14 | Denmark | 30 | Jamaica | 46 | Paraguay | 62 | Uruguay |
| 15 | Dominican Rep. | 31 | Japan | 47 | Peru | 63 | Venezuela, RB |
| 16 | Ecuador | 32 | Jordan | 48 | Philippines | 64 | Zimbabwe |

**Table B. Robustness Check for the 1991 Shock**

| **A:** Pre-Intervention Characteristics for TFR, 1980-1991   \|  \| China  (Actual) \| *Synth*China  (Simulated for Pre-Intervention Periods) \| Our Comparator  (All countries in the sample) \| \| --- \| --- \| --- \| --- \| \| *Pre-1991 but Post- 1979* \| \| \| \| \| TFR^[[1]](#footnote-1)^ \| 2.56 \| 2.57 \| 3.30 \| \| Male to Female (0-4) \| 1.07 \| 1.07 \| 1.05 \| \| Childbearing Age \| 26.27 \| 26.76 \| 28.14 \| \| Ln GDP per Capita \| 7.76 \| 8.85 \| 9.03 \| \| Life Expectancy at Birth \| 68.70 \| 70.13 \| 69.31 \| \| Years of Schooling \| 5.93 \| 6.69 \| 7.17 \| |
| --- | --- | --- | --- | --- | --- | --- | --- | --- | --- | --- | --- | --- | --- | --- | --- | --- | --- | --- | --- | --- | --- | --- | --- | --- | --- | --- | --- | --- | --- | --- | --- | --- |
| **B**: Countries Resembling China with Significant Weights for TFR, *Pre-1991 but Post- 1979*   \| Greece \| 68.3% \| \| --- \| --- \| \| India \| 31.2% \| \|  \|  \| |

**
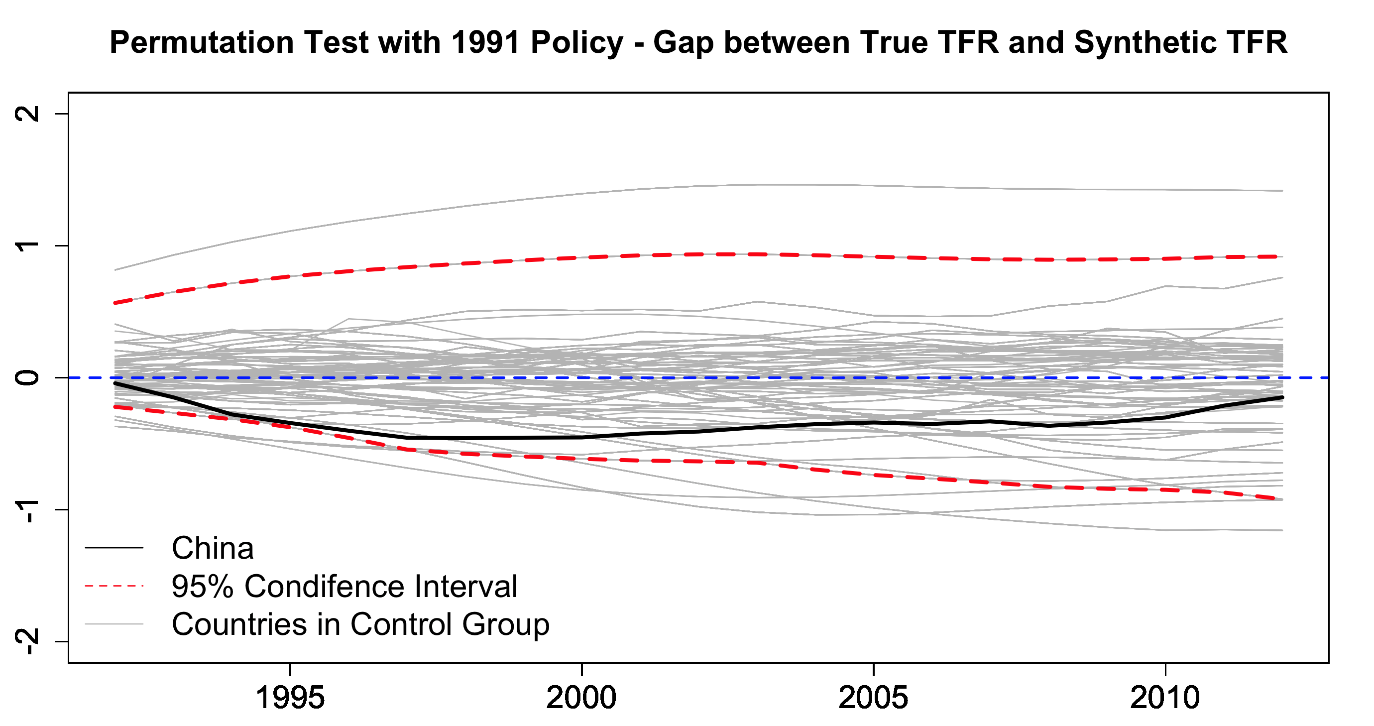
**

**Fig A. Robustness permutation test on the significance of the 1991 policy impact**

**Table C. Country List for Robustness Check using the Extended Dataset for the 1973 shock**

| 1 | Algeria | 37 | Guatemala | 73 | Pakistan |
| --- | --- | --- | --- | --- | --- |
| 2 | Argentina | 38 | Haiti | 74 | Panama |
| 3 | Australia | 39 | Honduras | 75 | Paraguay |
| 4 | Austria | 40 | HK SAR, China | 76 | Peru |
| 5 | Bangladesh | 41 | Iceland | 77 | Philippines |
| 6 | Barbados | 42 | India | 78 | Portugal |
| 7 | Belgium | 43 | Indonesia | 79 | Romania |
| 8 | Benin | 44 | Iran, Islamic Rep. | 80 | Rwanda |
| 9 | Bolivia | 45 | Ireland | 81 | Senegal |
| 10 | Botswana | 46 | Israel | 82 | Sierra Leone |
| 11 | Brazil | 47 | Italy | 83 | Singapore |
| 12 | Burundi | 48 | Jamaica | 84 | South Africa |
| 13 | Cameroon | 49 | Japan | 85 | Spain |
| 14 | Canada | 50 | Jordan | 86 | Sri Lanka |
| 15 | Central Africa Rep. | 51 | Kenya | 87 | Sweden |
| 16 | Chile | 52 | Korea, Rep. | 88 | Switzerland |
| 17 | China | 53 | Lesotho | 89 | Syrian Arab Rep. |
| 18 | Colombia | 54 | Liberia | 90 | Taiwan |
| 19 | Congo, Dem. Rep. | 55 | Luxembourg | 91 | Tanzania |
| 20 | Congo, Rep. | 56 | Malawi | 92 | Thailand |
| 21 | Costa Rica | 57 | Malaysia | 93 | Togo |
| 22 | Cote d'Ivoire | 58 | Mali | 94 | Trinidad & Tobago |
| 23 | Cyprus | 59 | Malta | 95 | Tunisia |
| 24 | Denmark | 60 | Mauritania | 96 | Turkey |
| 25 | Dominican Rep. | 61 | Mauritius | 97 | Uganda |
| 26 | Ecuador | 62 | Mexico | 98 | United Kingdom |
| 27 | Egypt, Arab Rep. | 63 | Morocco | 99 | United States |
| 28 | El Salvador | 64 | Mozambique | 100 | Uruguay |
| 29 | Fiji | 65 | Myanmar | 101 | Venezuela, RB |
| 30 | Finland | 66 | Namibia | 102 | Zambia |
| 31 | France | 67 | Nepal | 103 | Zimbabwe |
| 32 | Gabon | 68 | Netherlands |  |  |
| 33 | Gambia, The | 69 | New Zealand |  |  |
| 34 | Germany | 70 | Nicaragua |  |  |
| 35 | Ghana | 71 | Niger |  |  |
| 36 | Greece | 72 | Norway |  |  |

**Table D. Country List for Robustness Check using the Extended Dataset for the 1979 shock**

| 1 | Albania | 32 | Ecuador | 63 | Lesotho | 94 | Romania |
| --- | --- | --- | --- | --- | --- | --- | --- |
| 2 | Algeria | 33 | Egypt | 64 | Liberia | 95 | Rwanda |
| 3 | Argentina | 34 | El Salvador | 65 | Luxembourg | 96 | Saudi Arabia |
| 4 | Australia | 35 | Fiji | 66 | Macao SAR, China | 97 | Senegal |
| 5 | Austria | 36 | Finland | 67 | Malawi | 98 | Sierra Leone |
| 6 | Bahrain | 37 | France | 68 | Malaysia | 99 | Singapore |
| 7 | Bangladesh | 38 | Gabon | 69 | Maldives | 100 | South Africa |
| 8 | Barbados | 39 | Gambia, The | 70 | Mali | 101 | Spain |
| 9 | Belgium | 40 | Germany | 71 | Malta | 102 | Sri Lanka |
| 10 | Belize | 41 | Ghana | 72 | Mauritania | 103 | Sudan |
| 11 | Benin | 42 | Greece | 73 | Mauritius | 104 | Swaziland |
| 12 | Bolivia | 43 | Guatemala | 74 | Mexico | 105 | Sweden |
| 13 | Botswana | 44 | Haiti | 75 | Mongolia | 106 | Switzerland |
| 14 | Brazil | 45 | Honduras | 76 | Morocco | 107 | Syrian Arab Rep. |
| 15 | Brunei Darussalam | 46 | HK SAR, China | 77 | Mozambique | 108 | Taiwan |
| 16 | Bulgaria | 47 | Hungary | 78 | Myanmar | 109 | Tanzania |
| 17 | Burundi | 48 | Iceland | 79 | Namibia | 110 | Thailand |
| 18 | Cambodia | 49 | India | 80 | Nepal | 111 | Togo |
| 19 | Cameroon | 50 | Indonesia | 81 | Netherlands | 112 | Trinidad & Tobago |
| 20 | Canada | 51 | Iran | 82 | New Zealand | 113 | Tunisia |
| 21 | Central African Rep. | 52 | Iraq | 83 | Nicaragua | 114 | Turkey |
| 22 | Chile | 53 | Ireland | 84 | Niger | 115 | Uganda |
| 23 | China | 54 | Israel | 85 | Norway | 116 | United Arab Emirates |
| 24 | Colombia | 55 | Italy | 86 | Pakistan | 117 | United Kingdom |
| 25 | Congo, Dem. Rep. | 56 | Jamaica | 87 | Panama | 118 | United States |
| 26 | Congo, Rep. | 57 | Japan | 88 | Paraguay | 119 | Uruguay |
| 27 | Costa Rica | 58 | Jordan | 89 | Peru | 120 | Venezuela |
| 28 | Cote d'Ivoire | 59 | Kenya | 90 | Philippines | 121 | Vietnam |
| 29 | Cyprus | 60 | Korea, Rep. | 91 | Poland | 122 | Zambia |
| 30 | Denmark | 61 | Kuwait | 92 | Portugal | 123 | Zimbabwe |
| 31 | Dominican Rep. | 62 | Lao PDR | 93 | Qatar |  |  |

**Table E. Estimation Results of Synthetic Control Method Using Unbalanced Dataset**

| **A**: Pre-intervention characteristics   \|  \| China  (Actual) \| *Synth*China  (Simulated for Pre-Intervention Periods) \| Our Comparator  (All countries in the sample) \| \| --- \| --- \| --- \| --- \| \| *Pre- 1973: (103 countries)* \| \| \| \| \| TFR \| 5.85 \| 5.85 \| 5.30 \| \| Male to Female (0-4) \| 1.05 \| 1.05 \| 1.03 \| \| Childbearing Age \| 29.76 \| 29.73 \| 28.93 \| \| Ln GDP per Capita \| 7.12 \| 7.90 \| 8.22 \| \| Life Expectancy at Birth \| 56.44 \| 56.55 \| 57.68 \| \| Years of Schooling \| 3.67 \| 3.70 \| 4.02 \| \| *Pre-1979 but Post- 1973 (123 countries)* \| \| \| \| \| TFR \| 3.59 \| 3.63 \| 4.82 \| \| Male to Female (0-4) \| 1.06 \| 1.06 \| 1.03 \| \| Childbearing Age \| 29.17 \| 29.25 \| 28.76 \| \| Ln GDP per Capita \| 7.26 \| 8.19 \| 8.51 \| \| Life Expectancy at Birth \| 64.15 \| 61.43 \| 60.67 \| \| Years of Schooling \| 4.66 \| 4.69 \| 4.72 \| |
| --- | --- | --- | --- | --- | --- | --- | --- | --- | --- | --- | --- | --- | --- | --- | --- | --- | --- | --- | --- | --- | --- | --- | --- | --- | --- | --- | --- | --- | --- | --- | --- | --- | --- | --- | --- | --- | --- | --- | --- | --- | --- | --- | --- | --- | --- | --- | --- | --- | --- | --- | --- | --- | --- | --- | --- | --- | --- | --- | --- | --- |
| **B**: Countries Resembling China with Significant Weights   \| Pre-1973 Period^[[2]](#footnote-2)^ \| \| Pre-1979 but Post- 1973 Period^[[3]](#footnote-3)^ \| \| \| --- \| --- \| --- \| --- \| \| India \| 29.9% \| India \| 27.9% \| \| Ireland \| 13.6% \| Korea \| 31.7% \| \| Jordan \| 22.8% \| Macao SAR, China \| 22.8% \| \| Korea \| 8.8% \| Nepal \| 1.0% \| \| Pakistan \| 1.0% \|  \|  \| |

| **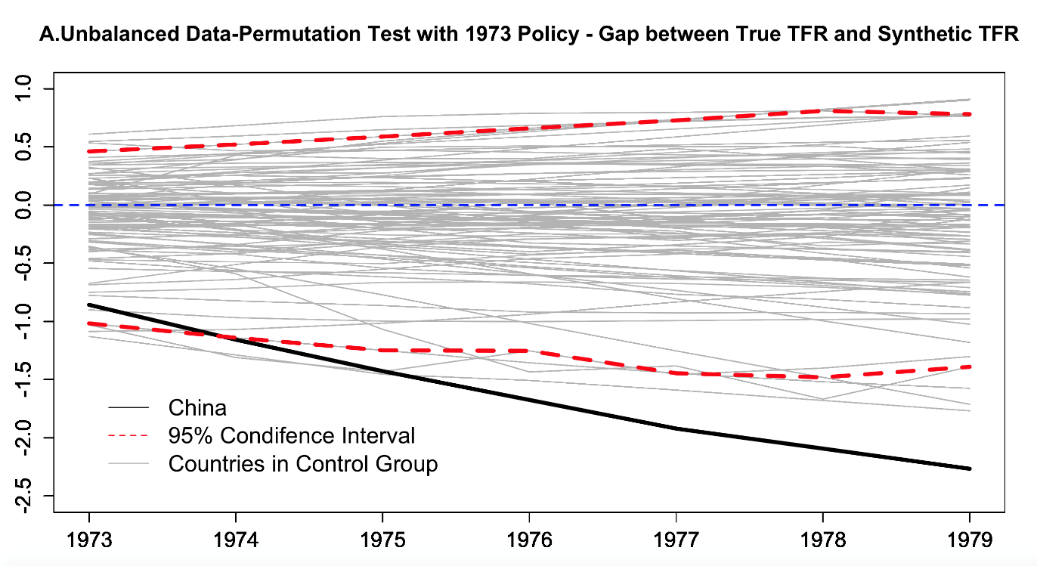** |
| --- |
| **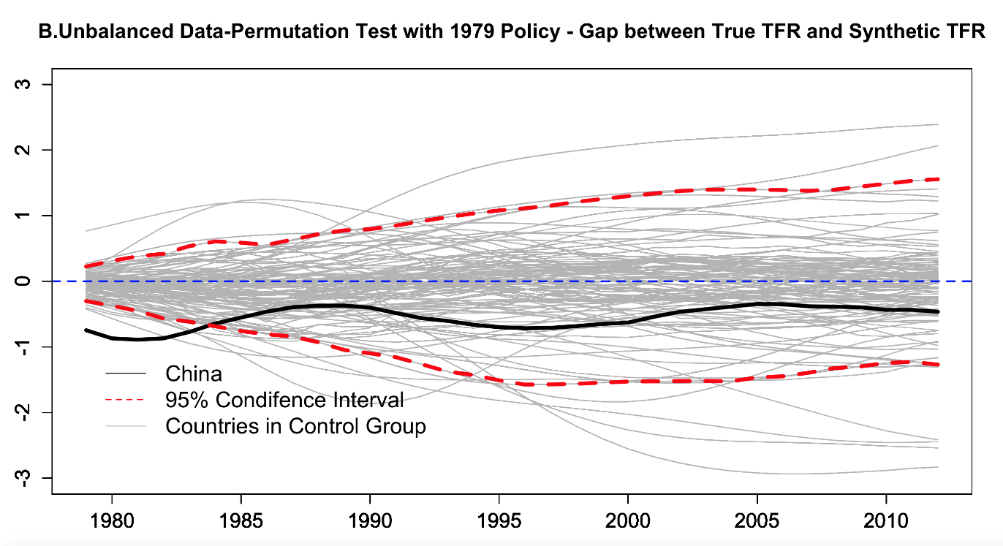** |

**Fig B. Robustness permutation test on the significance of the policy impact using unbalanced Data**

**Table F. Country List for Robustness Check Using the Selected Dataset**

| Selected 25 Countries as the Dataset | | | | | | | |  |
| --- | --- | --- | --- | --- | --- | --- | --- | --- |
| 1 | Albania | 8 | Panama | 15 | India | 22 | Indonesia |  |
| 2 | Brazil | 9 | Paraguay | 16 | Japan | 23 | Vietnam |  |
| 3 | Colombia | 10 | South Africa | 17 | Korea, Rep. | 24 | Malaysia |  |
| 4 | Costa Rica | 11 | Turkmenistan | 18 | Pakistan | 25 | Mongolia |  |
| 5 | Jamaica | 12 | Uzbekistan | 19 | Philippines |  |  |  |
| 6 | Korea, Dem. Peop. Rep. | 13 | Venezuela | 20 | Taiwan |  |  |  |
| 7 | Lebanon | 14 | China | 21 | Thailand |  |  |  |
| For the 1973 Shock | | | | | | | | |
| 1 | China | 6 | Jamaica | 11 | Panama | 16 | Thailand |  |
| 2 | Colombia | 7 | Japan | 12 | Paraguay | 17 | Venezuela |  |
| 3 | Costa Rica | 8 | Korea, Rep. | 13 | Philippines |  |  |  |
| 4 | India | 9 | Malaysia | 14 | South Africa |  |  |  |
| 5 | Indonesia | 10 | Pakistan | 15 | Taiwan |  |  |  |
| For the 1979 Shock | | | | | | | | |
| 1 | Albania | 6 | Indonesia | 11 | Mongolia | 16 | South Africa |  |
| 2 | China | 7 | Jamaica | 12 | Pakistan | 17 | Taiwan |  |
| 3 | Colombia | 8 | Japan | 13 | Panama | 18 | Thailand |  |
| 4 | Costa Rica | 9 | Korea, Rep. | 14 | Paraguay | 19 | Venezuela |  |
| 5 | India | 10 | Malaysia | 15 | Philippines | 20 | Vietnam |  |

**Table G. Estimation Results of Synthetic Control Method Using Selected Countries**

| \| **A**: Pre-intervention characteristics   \|  \| China  (Actual) \| *Synth*China  (Simulated for Pre-Intervention Periods) \| Our Comparator  (All countries in the sample) \| \| --- \| --- \| --- \| --- \|   *Pre- 1973: (17 countries)* \| \| \| \| \| \| --- \| --- \| --- \| --- \| --- \| --- \| --- \| --- \| --- \| \| TFR \| 5.85 \| \| 5.80 \| 5.63 \| \| Male to Female (0-4) \| 1.05 \| \| 1.04 \| 1.04 \| \| Childbearing Age \| 29.76 \| \| 29.73 \| 29.24 \| \| Ln GDP per Capita \| 7.12 \| \| 7.30 \| 8.11 \| \| Life Expectancy at Birth \| 56.44 \| \| 56.40 \| 61.06 \| \| Years of Schooling \| 3.67 \| \| 2.48 \| 4.08 \| \| *Pre-1979 but Post- 1973 (20 countries)* \| \| \| \| \| \| TFR \| \| 3.59 \| 3.62 \| 4.62 \| \| Male to Female (0-4) \| \| 1.06 \| 1.06 \| 1.04 \| \| Childbearing Age \| \| 29.17 \| 29.35 \| 29.19 \| \| Ln GDP per Capita \| \| 7.26 \| 8.22 \| 8.25 \| \| Life Expectancy at Birth \| \| 64.15 \| 63.65 \| 63.89 \| \| Years of Schooling \| \| 4.66 \| 5.83 \| 4.97 \| |
| --- | --- | --- | --- | --- | --- | --- | --- | --- | --- | --- | --- | --- | --- | --- | --- | --- | --- | --- | --- | --- | --- | --- | --- | --- | --- | --- | --- | --- | --- | --- | --- | --- | --- | --- | --- | --- | --- | --- | --- | --- | --- | --- | --- | --- | --- | --- | --- | --- | --- | --- | --- | --- | --- | --- | --- | --- | --- | --- | --- | --- | --- | --- | --- | --- | --- | --- | --- | --- | --- | --- | --- | --- | --- | --- |
| **B**: Countries Resembling China with Significant Weights   \| Pre-1973 Period^[[4]](#footnote-4)^ \| \| Pre-1979 but Post- 1973 Period^[[5]](#footnote-5)^ \| \| \| --- \| --- \| --- \| --- \| \|  \|  \|  \|  \| \| India \| 8.5% \| Korea \| 64.10% \| \| Indonesia \| 30.3% \| Thailand \| 35.70% \| \| Thailand \| 59.8% \|  \|  \| |

| **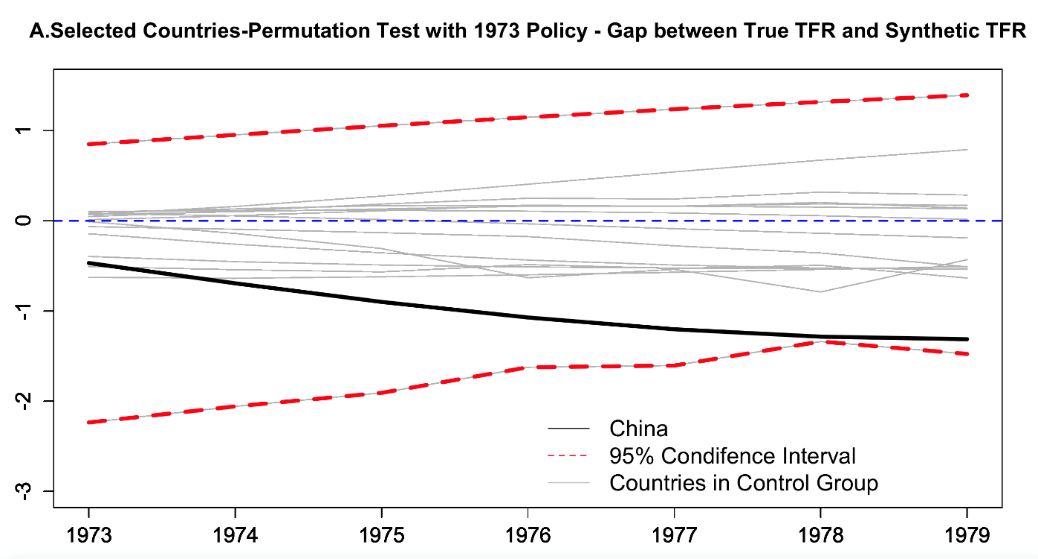** |
| --- |
| **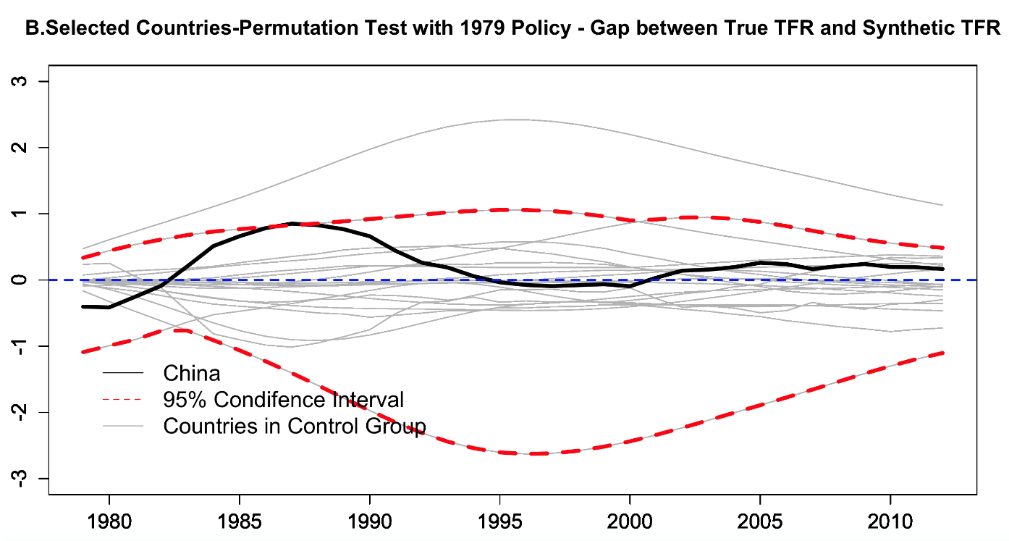** |

**Fig C. Robustness permutation test on the significance of the policy impact for selected countries**

**Table H. Comparison of TFRs used in our analysis and UN Interpolated TFRs**

|  | TFR | TFR (UN) |
| --- | --- | --- |
| Minimum | 0.83 | 0.83 |
| 1 Quartile | 2.33 | 2.33 |
| Median | 4.21 | 4.21 |
| Mean | 4.29 | 4.30 |
| 3rd Quartile | 6.21 | 6.25 |
| Maximum | 8.45 | 8.46 |


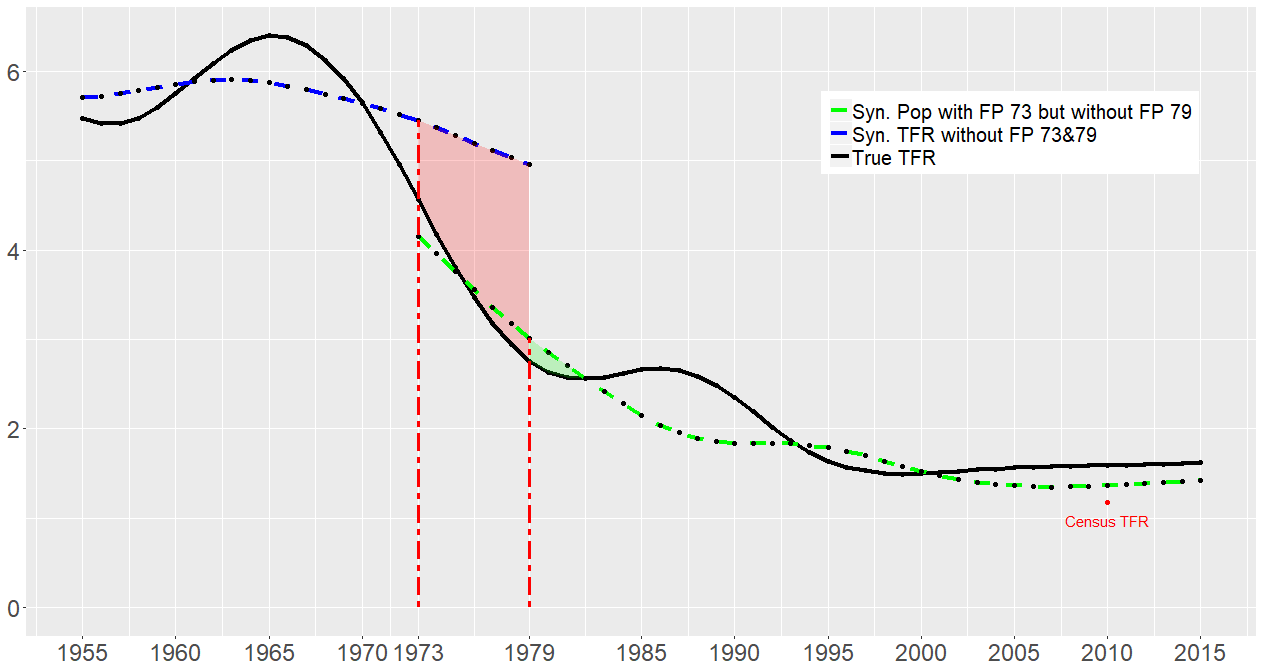


**Fig D. Time trend of ‘actual’ and ‘synthetic’ Total Fertility Rate for China using UN interpolated data, 1955-2015**

1. The pre-intervention period for TFR is 1980-1991 while for the others are 1985-1991. [↑](#footnote-ref-1)
2. Altogether, 57 countries were used to construct the *Synth*China. Here we only present the countries with weights higher than 1%. [↑](#footnote-ref-2)
3. Altogether, 21 countries were used to construct the *Synth*China. Here we only present the countries with weights higher than 1%. [↑](#footnote-ref-3)
4. Altogether, 57 countries were used to construct the *Synth*China. Here we only present the countries with weights higher than 1%. [↑](#footnote-ref-4)
5. Altogether, 21 countries were used to construct the *Synth*China. Here we only present the countries with weights higher than 1%. [↑](#footnote-ref-5)
